# Supplementary material for: Physiological DNA damage promotes functional endoreplication of mammary gland alveolar cells during lactation
Source: Nat Commun. 2024 Apr 17;15:3288. doi: 10.1038/s41467-024-47668-9 (PMC11021458; doi:10.1038/s41467-024-47668-9)
Supplement: Supplementary file 1 — Supplementary Information [file 41467_2024_47668_MOESM1_ESM.pdf]

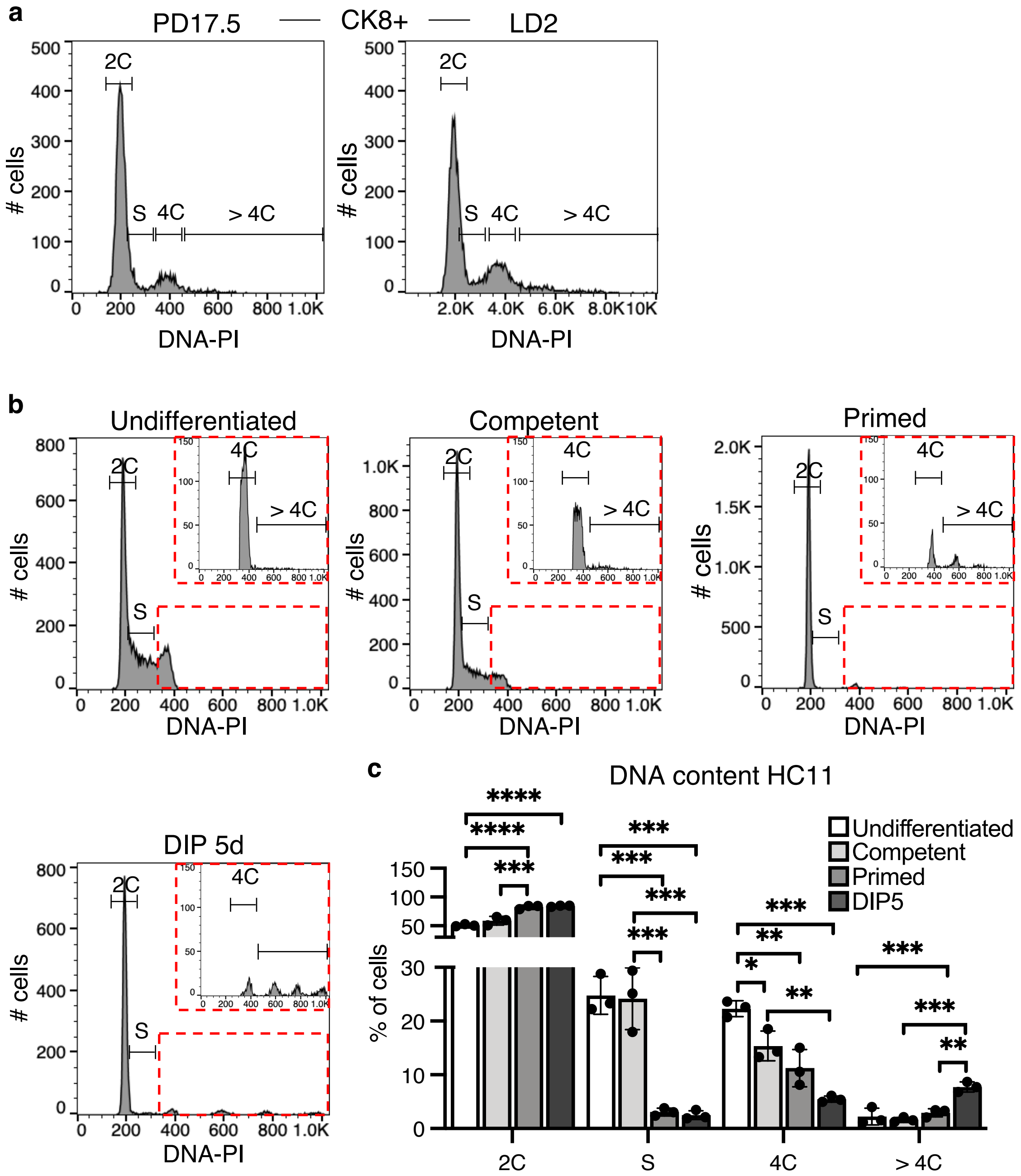

Supplementary Figure 1. Endoreplication occurs during lactogenic differentiation.
a) Representative FACS histograms for DNA content analysis of the CK8+ population in PD17.5 (left) and LD2 MGs (right). **b)** Representative FACS histograms for DNA content analysis of undifferentiated, competent, primed and differentiated (DIP5) HC11 cells. Red dashed lines show a magnification of the histogram corresponding to the 4C and >4C DNA content populations. **c)** Percentage of undifferentiated, competent, primed and differentiated (DIP5) HC11 cells with 2C, 4C or > 4C DNA content or in the S phase, as detected by FACS analysis. Data presented as mean ± SD. Data analyzed by one-way ANOVA with Tukey's multiple comparison test. Data representative of n=3 biologically independent experiments. p values: * < 0.05, **< 0.01, *** < 0.001, **** < 0.0001.

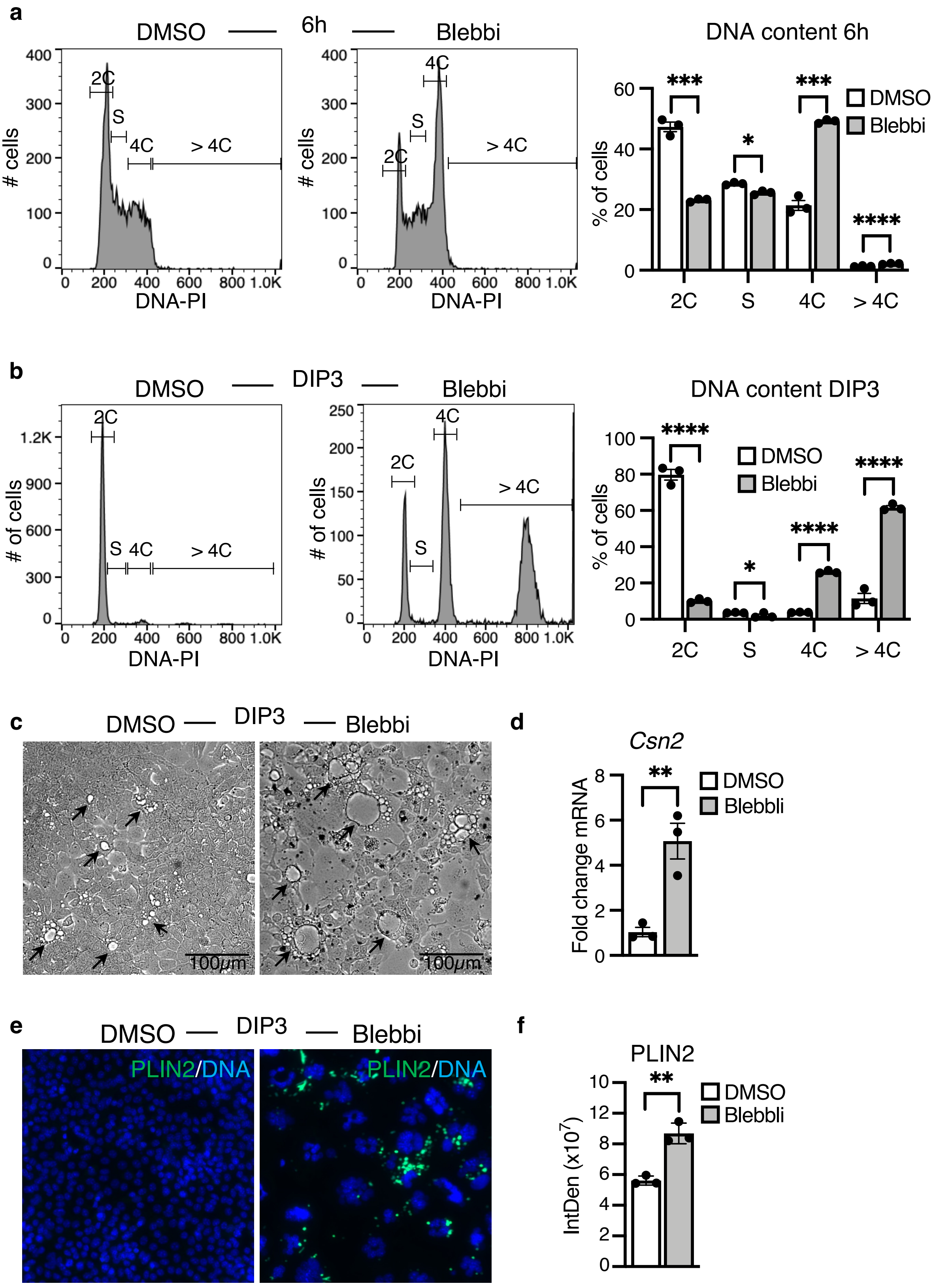
a
DMSO — 6h — Blebbi
400
300
200
100
0
cells
2C
S
4C
> 4C
0 200 400 600 800 1.0K
DNA-PI
4C
2C
S
> 4C
DNA content 6h
60
40
20
0
% of cells
***
*
***
****
DMSO
Blebbi
2C S 4C > 4C
b
DMSO — DIP3 — Blebbi
1.2K
900
600
300
0
# of cells
2C
S 4C > 4C
0 200 400 600 800 1.0K
DNA-PI
250
200
150
100
50
0
4C
2C
S
> 4C
DNA content DIP3
100
80
60
40
20
0
% of cells
****
*
****
****
DMSO
Blebbi
2C S 4C > 4C
c
DMSO — DIP3 — Blebbi
100μm
100μm
d
Csn2
8
6
4
2
0
Fold change mRNA
**
DMSO
Blebbli
e
DMSO — DIP3 — Blebbi
PLIN2/DNA
PLIN2/DNA
f
PLIN2
10
12
8
6
4
2
0
IntDen (x10^7)
**
DMSO
Blebbli

Supplementary Figure 2. Cytokinesis failure results in endoreplication and increased milk production. a) Representative FACS histograms for DNA content analysis (left) and quantification (right) of undifferentiated HC11 cells treated with DMSO or blebbistatin (Blebbi) for 6 hours. **b)** Representative FACS histograms for DNA content analysis (left) and quantification (right) of differentiated (DIP3) HC11 cells treated with DMSO or Blebbi. **c)** Representative phase contrast images of showing milk domes in differentiated (DIP3) HC11 cells, treated with DMSO or Blebbi. **d)** *Csn2* expression in differentiated (DIP3) HC11 cells, treated with DMSO or Blebbi, as detected by RT-qPCR. **e)** Representative images of Perilipin2 (PLIN2, green) in differentiated (DIP3) HC11 cells, treated with DMSO or Blebbi. Nuclear DNA by Hoechst. **f)** Quantification of PLIN2 in differentiated (DIP3) HC11 cells, treated with DMSO or Blebbi, as detected by IHC. Data presented as mean ± SD **(a, b and d)** and mean ± S.E.M **(f)**. Data analyzed by unpaired, two-tailed Student's t-test. Data representative of n=3 biologically independent experiments. p values: * < 0.05, **< 0.01, *** < 0.001, **** < 0.0001.

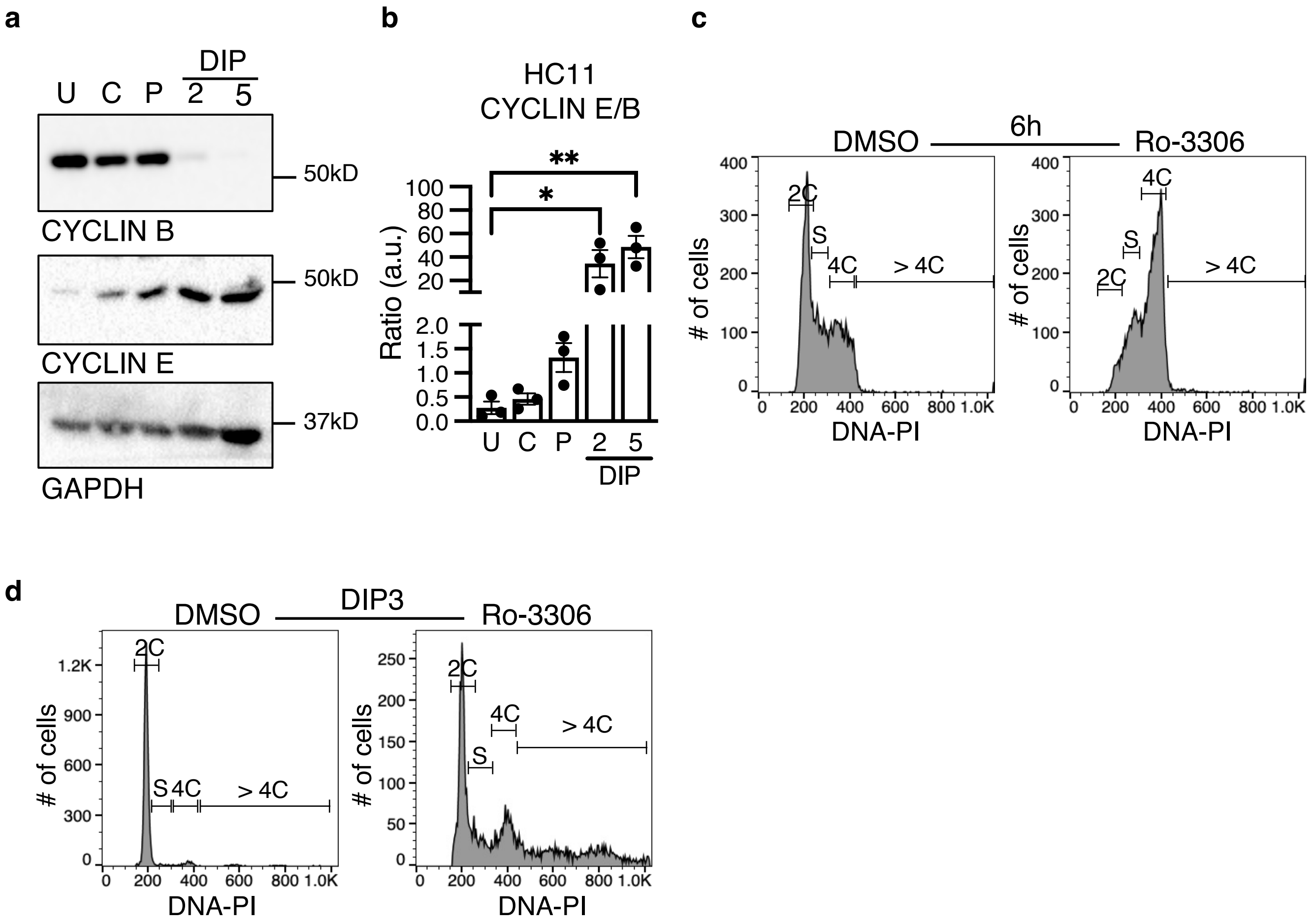

Supplementary Figure 3. HC11 cells undergo endoreplication through an early mitotic arrest involving Cdk1 inactivation. a and b) Representative western blot **(a)** and quantification **(b)** of CYCLIN E and CYCLIN B expression in undifferentiated (U), competent (C), primed (P) and differentiated (DIP 2 and 5) HC11 cells. Quantification **(b)** shown as CYCLIN E/CYCLIN B ratio. **c)** Representative FACS histograms for DNA content analysis of undifferentiated HC11 cells treated with DMSO or Ro-3306 for 6 hours. **d)** Representative FACS histograms for DNA content analysis of differentiated (DIP3) HC11 cells treated with DMSO or Ro-3306. Data presented as mean ± S.E.M. Data analyzed by one-way ANOVA with Tukey's multiple comparison test. Data representative of n=3 biologically independent experiments. p values: * < 0.05, **< 0.01.

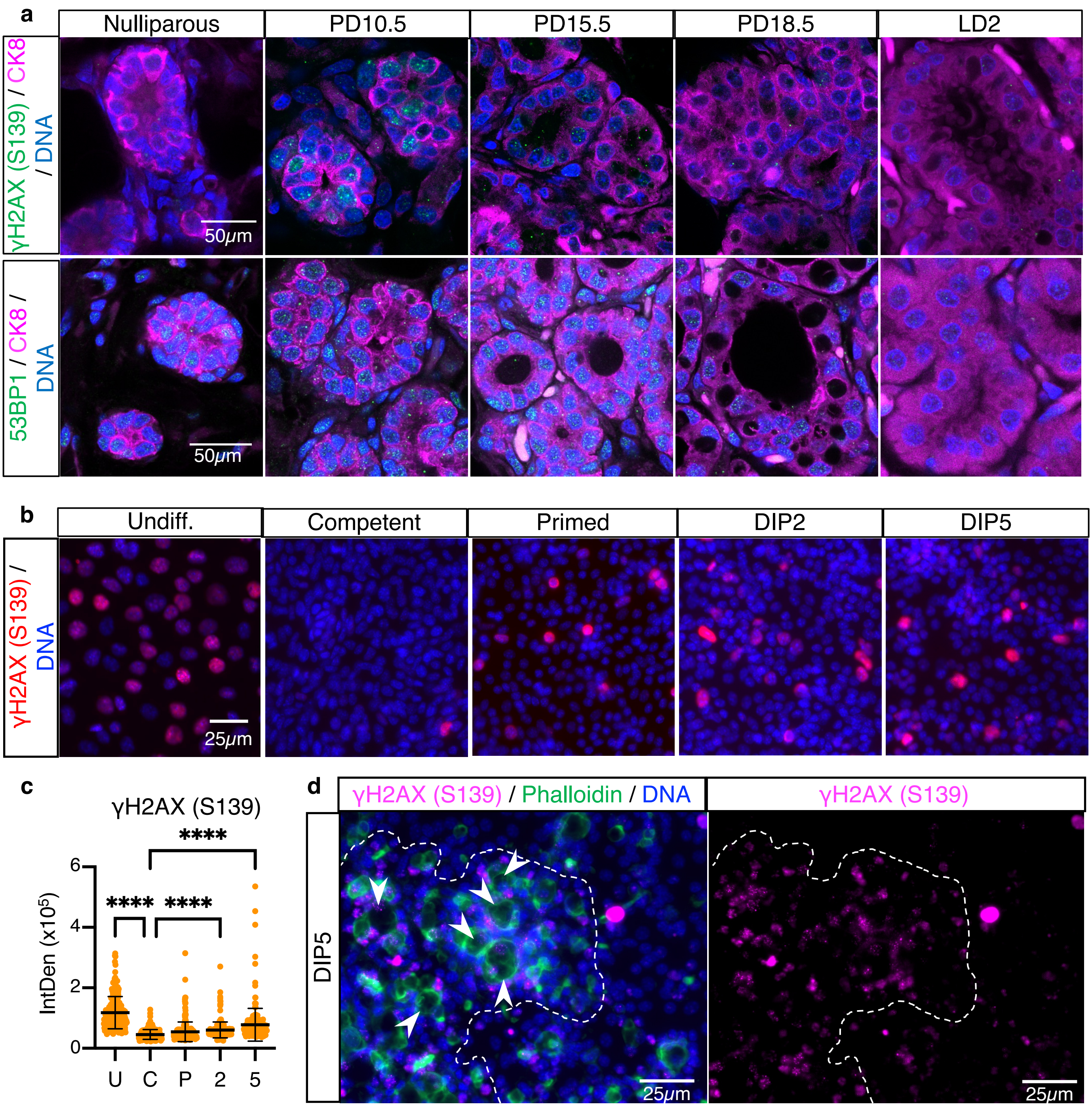
a
Nulliparous
PD10.5
PD15.5
PD18.5
LD2
γH2AX (S139) / CK8 / DNA
53BP1 / CK8 / DNA
50μm
50μm
b
Undiff.
Competent
Primed
DIP2
DIP5
γH2AX (S139) / DNA
25μm
c
γH2AX (S139)
IntDen (x10^5)
****
****
****
6
4
2
0
U
C
P
2
5
d
γH2AX (S139) / Phalloidin / DNA
γH2AX (S139)
DIP5
25μm
25μm

Supplementary Figure 4. Physiological DNA damage occurs during alveologenesis. **a)** Representative images of γH2AX (green, top) or 53BP1 (green, bottom) in optically-cleared sections of nulliparous, PD10.5, PD15.5, PD18.5 and LD2 MGs. CK8 shown in magenta. Nuclear DNA by propidium iodide. **b)** Representative images of γH2AX (red) in undifferentiated, competent, primed and differentiated (DIP 2 and 5) HC11 cells. Nuclear DNA by Hoechst. **c)** Quantification of nuclear γH2AX in HC11 cells throughout differentiation, as detected by ICC. **d)** Representative images of differentiated (DIP5) HC11 cells showing that γH2AX (magenta) is high in areas with dome formation (white dashed line, Phalloidin, green) Domes are indicated by arrows. Nuclear DNA by Hoechst. For **(c)**, data show values of 250 cells per time-point (orange dots). Data presented as mean ± S.E.M. Data analyzed by one-way ANOVA with Dunn's multiple comparison test. Data representative of n=3 biologically independent experiments. p values: **** < 0.0001.

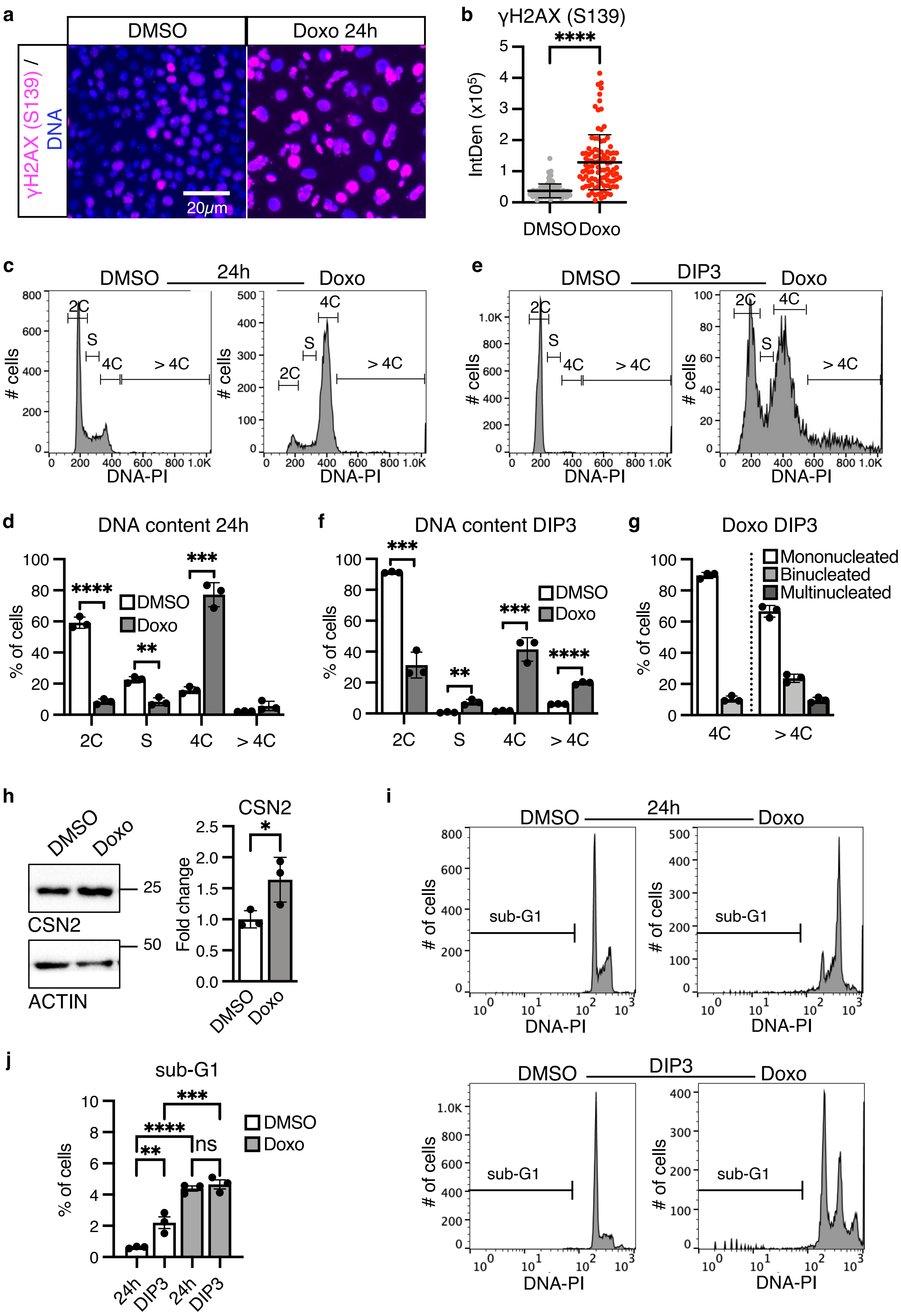
a
DMSO
Doxo 24h
γH2AX (S139) / DNA
20μm
b
γH2AX (S139)
IntDen (x10^5)
****
DMSO
Doxo
c
DMSO
24h
Doxo
cells
2C
S
4C
> 4C
DNA-PI
e
DMSO
DIP3
Doxo
d
DNA content 24h
% of cells
DMSO
Doxo
2C
S
4C
> 4C
f
DNA content DIP3
g
Doxo DIP3
Mononucleated
Binucleated
Multinucleated
h
CSN2
ACTIN
25
50
Fold change
*
i
sub-G1
# of cells
j
sub-G1
ns
24h
DIP3

Supplementary Figure 5. DNA damage increases endoreplication and milk production *in vitro*. a) Representative images of γH2AX (magenta) in undifferentiated HC11 cells treated with DMSO or Doxo for 24 hours. Nuclear DNA by Hoechst. **b)** Quantification of nuclear γH2AX in HC11 cells 24 hours after treatment with DMSO or Doxo, as detected by ICC. **c)** Representative FACS histogram for DNA content analysis of undifferentiated HC11 cells 24 hours after treatment with DMSO or Doxo. **d)** Percentage of undifferentiated HC11 cells with 2C, 4C or > 4C DNA content, or in the S phase, as detected by FACS analysis, 24 hours after treatment with DMSO or Doxo. **e)** Representative FACS histograms for DNA content analysis of differentiated (DIP3) HC11 cells treated with DMSO or Doxo. **f)** Percentage of differentiated (DIP3) HC11 cells with 2C, 4C or > 4C DNA content, or in the S phase, as detected by FACS analysis, after treatment with DMSO or Doxo. **g)** Quantification of differentiated (DIP3) HC11 cells treated with DMSO or Doxo and sorted based on DNA content (4C or > 4C), that are mono-, bi- or multinucleated (more than 2 nuclei). **h)** Representative Western Blot (left) and quantification (right) of CSN2 expression in differentiated (DIP3) HC11 cells after DMSO or Doxo treatment. **i)** Representative FACS histograms for DNA content analysis showing the sub-G1 population of HC11 cultures treated with DMSO or doxorubicin (Doxo) for 24h (top histograms) or after 3 days of differentiation (DIP3, bottom histograms). **j)** Percentage of HC11 cells within the sub-G1 population, as detected by FACS analysis, 24h after treatment with DMSO or Doxo, or 3 days after differentiation (DIP3). For **(b)** data show values of 100 cells per treatment (gray or red dots). Data presented as mean ± SEM **(b)** and mean ± SD **(c, d, f-h and j)**. Data analyzed by Mann-Whitney u-test **(b)** and unpaired, two-tailed Student's t-test **(c, d, f-h and j)**. Data representative of n=3 biologically independent experiments. p values: * < 0.05, **< 0.01, *** < 0.001, **** < 0.0001.

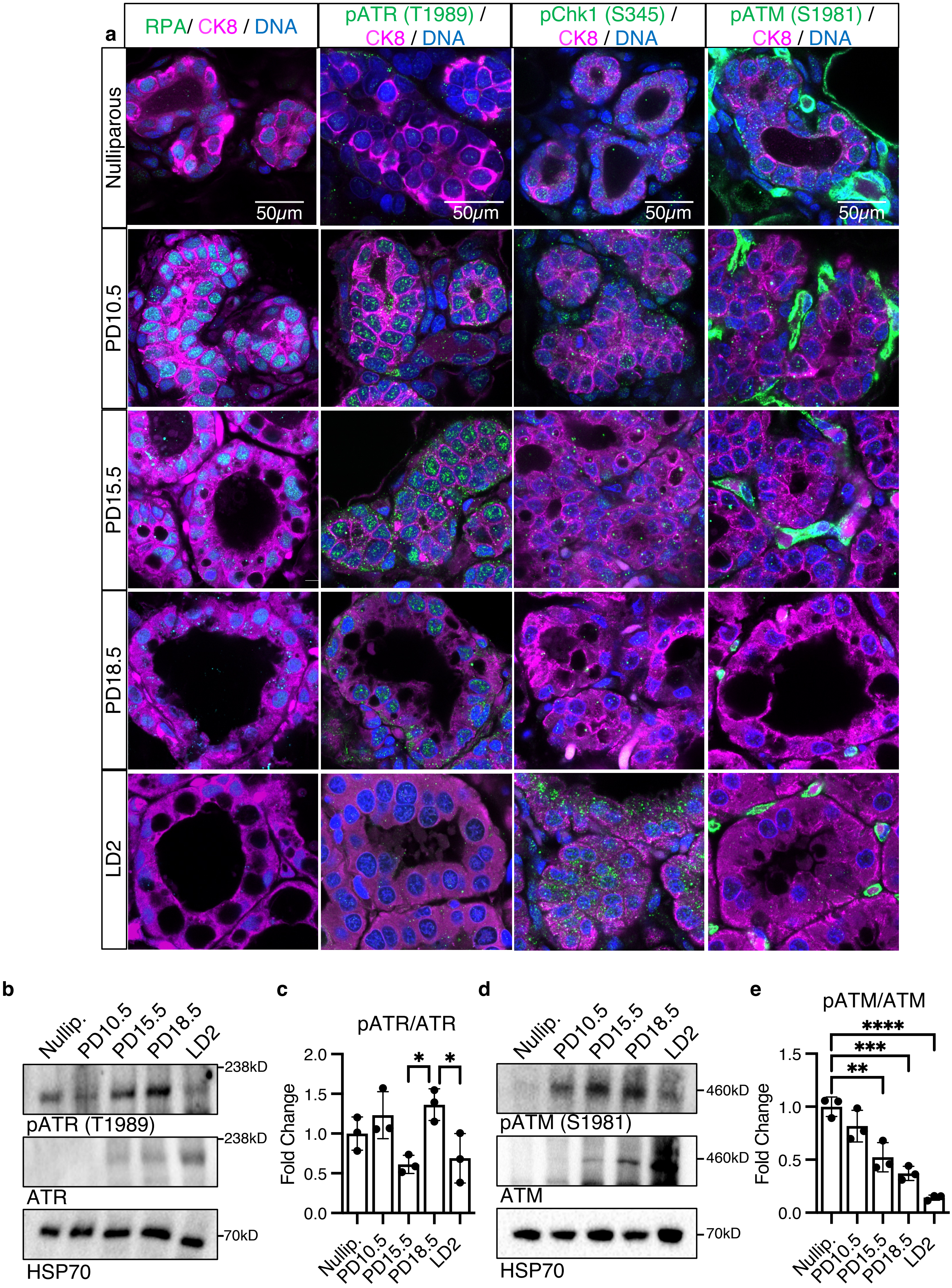
a
RPA/ CK8 / DNA
pATR (T1989) / CK8 / DNA
pChk1 (S345) / CK8 / DNA
pATM (S1981) / CK8 / DNA
Nulliparous
PD10.5
PD15.5
PD18.5
LD2
50μm
b
Nullip.
PD10.5
PD15.5
PD18.5
LD2
238kD
pATR (T1989)
ATR
70kD
HSP70
c
pATR/ATR
Fold Change
2.0
1.5
1.0
0.5
0.0
*
d
460kD
pATM (S1981)
ATM
e
pATM/ATM
****
***
**

Supplementary Figure 6. The DNA damage response to replication stress is activated during pregnancy and lactation. a) Representative images of RPA, pATR, pCHK1 or pATM (green) in optically-cleared sections of nulliparous, PD10.5, PD15.5, PD18.5 and LD2 MGs. CK8 shown in magenta. Nuclear DNA by propidium iodide. **b and c)** Representative western blot **(b)** and quantification **(c)** of pATR and ATR expression in nulliparous, PD10.5, PD15.5, PD18.5 and LD2 MGs. Quantification **(c)** shown as pATR/ATR ratio. **d and e)** Representative western blot **(d)** and quantification **(e)** of pATM and ATM expression in nulliparous, PD10.5, PD15.5, PD18.5 and LD2 MGs. Quantification **(e)** shown as pATM/ATM ratio. Data presented as mean ± SD. Data analyzed by one-way ANOVA with Tukey's multiple comparison test. Data representative of n=3 biologically independent experiments. p values: * < 0.05, **< 0.01, *** < 0.001.

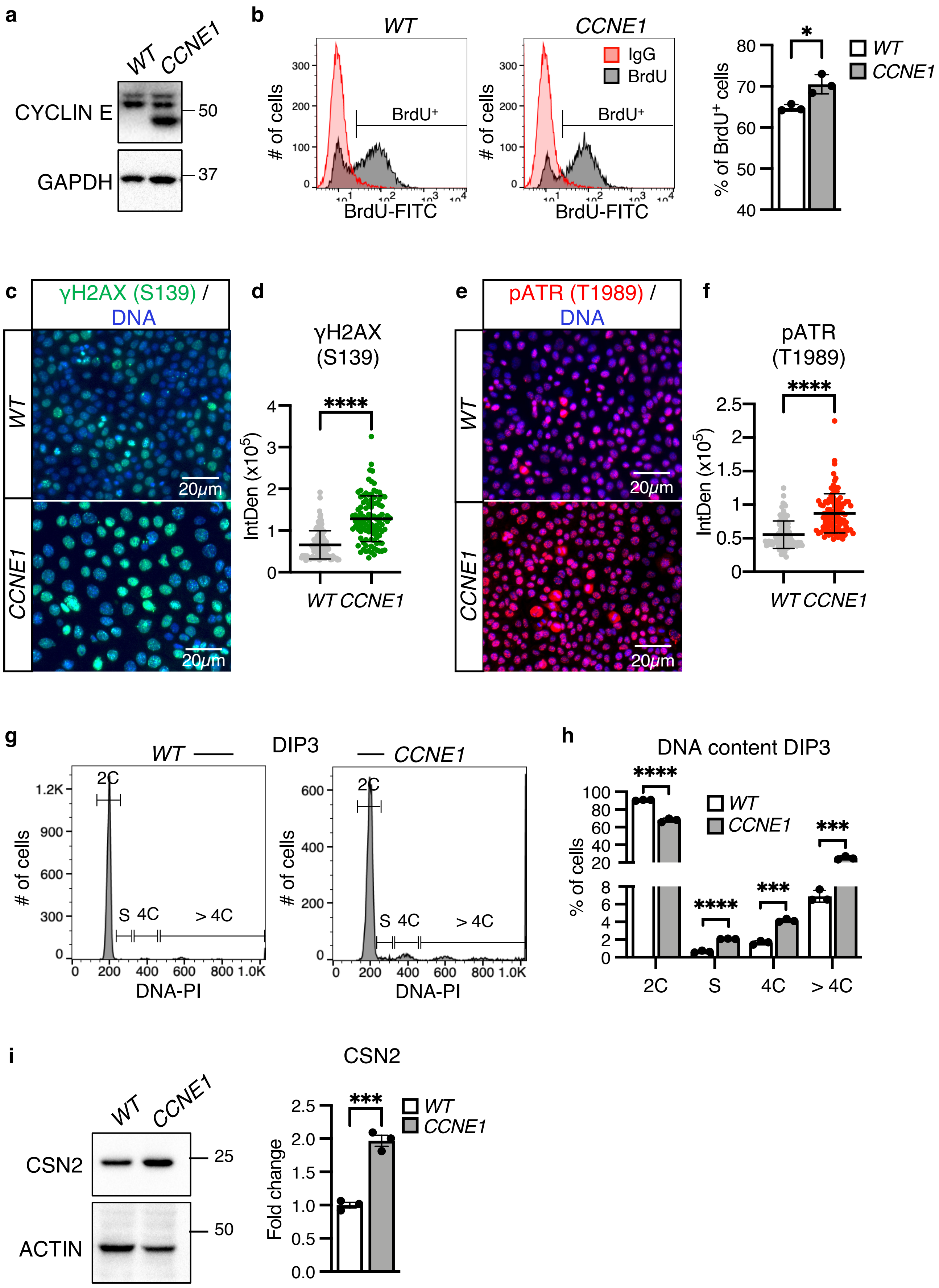

a
WT
CCNE1
CYCLIN E
50
GAPDH
37
b
WT
CCNE1
IgG
BrdU
BrdU+
# of cells
BrdU-FITC
% of BrdU+ cells
*
c
γH2AX (S139) / DNA
WT
CCNE1
20μm
d
γH2AX (S139)
IntDen (x10^5)
****
e
pATR (T1989) / DNA
f
pATR (T1989)
g
DIP3
2C
S 4C > 4C
DNA-PI
h
DNA content DIP3
% of cells
2C
S
4C
> 4C
****
***
i
CSN2
ACTIN
25
50
Fold change
***

Supplementary Figure 7. Replication stress activates the DNA damage response and results in increased endoreplication and milk production *in vitro.* a) Detection of CYCLIN E by Western blot in wildtype (*WT*) or Cyclin E overexpressing (*CCNE1*) HC11 cells. **b)** Representative FACS histograms showing BrdU incorporation in *WT* and *CCNE1* undifferentiated HC11 cells. Red histogram represents the negative isotype control (IgG). Black gate represents the identification of the $BrdU^+$ cells according to negative isotype control. Right histogram shows the quantification of *WT* or *CCNE1* HC11 cells that are $BrdU^+$. **c and d)** Representative images **(c)** and quantification **(d)** of nuclear γH2AX (green) in undifferentiated WT or *CCNE1* HC11 cells. Nuclear DNA by Hoechst. **e and f)** Representative images **(e)** and quantification **(f)** of nuclear pATR (red) in undifferentiated *WT* or *CCNE1* HC11 cells. Nuclear DNA by Hoechst. **g)** Representative FACS histograms for DNA content analysis of *WT* or *CCNE1* differentiated (DIP3) HC11 cells. **h)** Percentage of differentiated (DIP3) *WT* or *CCNE1* HC11 cells with 2C, 4C or >4C DNA content, or in the S phase, as detected by FACS analysis. **i)** Representative western blot (left) and quantification (right) of CSN2 expression in differentiated (DIP3) *WT* or *CCNE1* HC11 cells. For **(d and f)**, data show values of 100 cells per treatment (gray, green or red dots). Data presented as mean ± SD **(b, h and i)** and mean ± SEM **(d and f)**. Data analyzed by unpaired, two-tailed Student's t-test **(b, h and i)** and Mann-Whitney u-test **(d and f)**. Data representative of n=3 biologically independent experiments. p values: * < 0.05, *** < 0.001, **** < 0.0001.

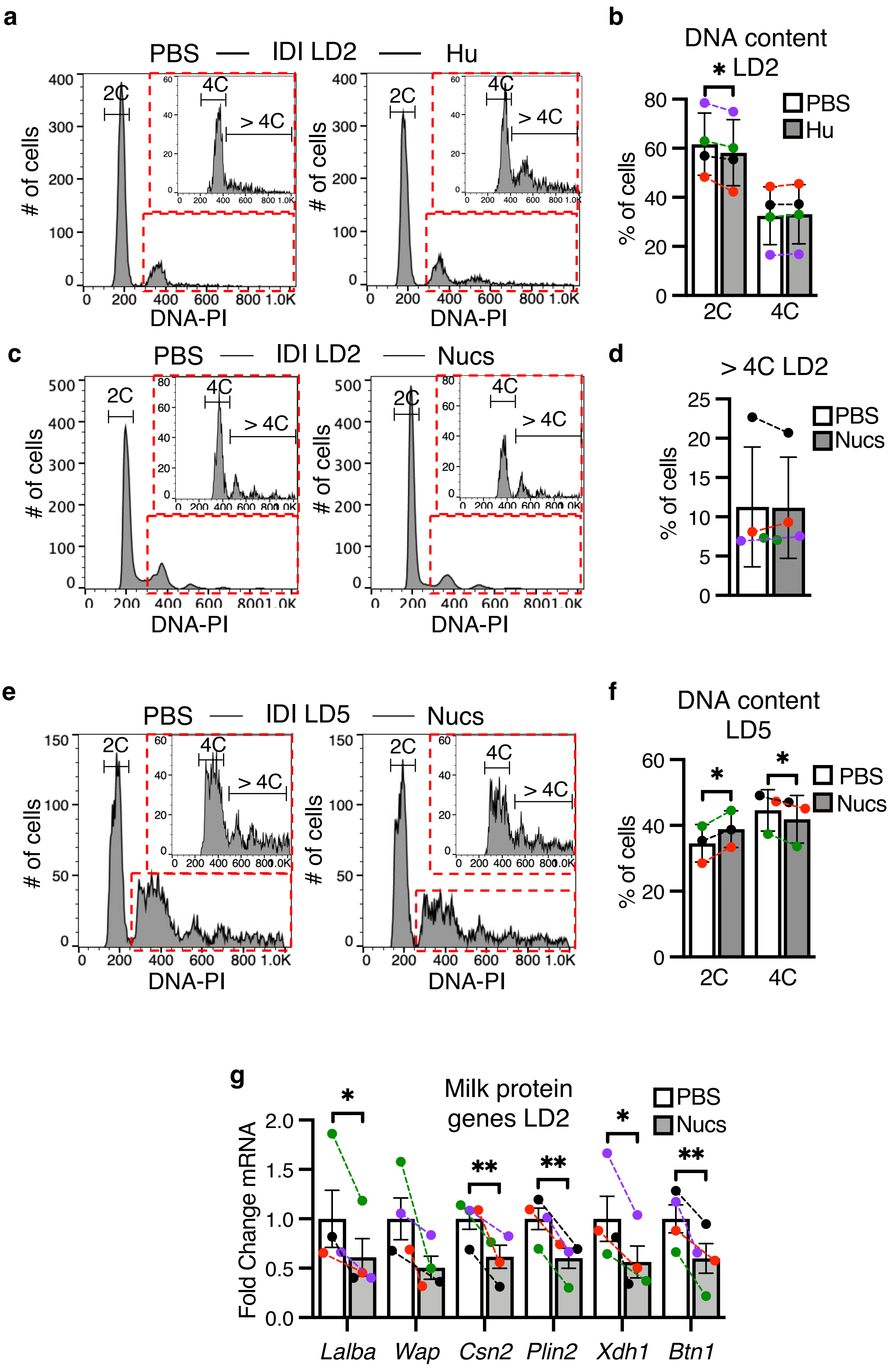
a
PBS — IDI LD2 — Hu
2C
4C
> 4C
# of cells
DNA-PI
b
DNA content
* LD2
PBS
Hu
% of cells
2C
4C
c
PBS — IDI LD2 — Nucs
2C
4C
> 4C
# of cells
DNA-PI
d
> 4C LD2
PBS
Nucs
% of cells
e
PBS — IDI LD5 — Nucs
2C
4C
> 4C
# of cells
DNA-PI
f
DNA content
LD5
PBS
Nucs
% of cells
2C
4C
g
Milk protein
genes LD2
PBS
Nucs
Fold Change mRNA
Lalba
Wap
Csn2
Plin2
Xdh1
Btn1

Supplementary Figure 8. Replication stress regulates endoreplication and milk production *in vivo.* a) Representative FACS DNA content analysis histograms from $CK8^+$ LD2 MGs after contralateral IDI with PBS or hydroxyurea (Hu). Red dashed lines show a magnification of the histogram corresponding to the 4C and >4C DNA content populations. **b)** Percentage of $CK8^+$ cells with 2C or 4C DNA content in LD2 MGs, as detected by FACS analysis, after contralateral IDI with PBS or Hu. Colored data points and dashed lines represent paired samples. **c)** Representative FACS DNA content analysis histograms from $CK8^+$ LD2 MGs after contralateral IDI with PBS or nucleosides (Nucs). Red dashed lines show a magnification of the histogram corresponding to the 4C and >4C DNA content populations. **d)** Percentage of $CK8^+$ cells with > 4C DNA content in LD2 MGs, as detected by FACS analysis, after contralateral IDI with PBS or Nucs. Colored data points and dashed lines represent paired samples. **e)** Representative FACS DNA content analysis histograms from $CK8^+$ LD5 MGs after contralateral IDI with PBS or nucleosides (Nucs). Red dashed lines show a magnification of the histogram corresponding to the 4C and >4C DNA content populations. **f)** Percentage of $CK8^+$ cells with 2C or 4C DNA content in LD5 MGs, as detected by FACS analysis, after contralateral IDI with PBS or Nucs. Colored data points and dashed lines represent paired samples. **g)** Milk protein gene expression, *Lalba, Wap, Csn2, Plin2, Xdh1* and *Btn1*, in LD2 MGs after contralateral IDI with PBS or Nucs, as detected by RT-qPCR. Colored data points and dashed lines represent paired samples. Data presented as mean ± SD **(b, d and f)** and mean ± SEM **(g)**. Data analyzed by paired, two-tailed Student's t-test. Data representative of n=4 biologically independent experiments except **(e and f)**, representative of n=3. p values: * < 0.05, **< 0.01.

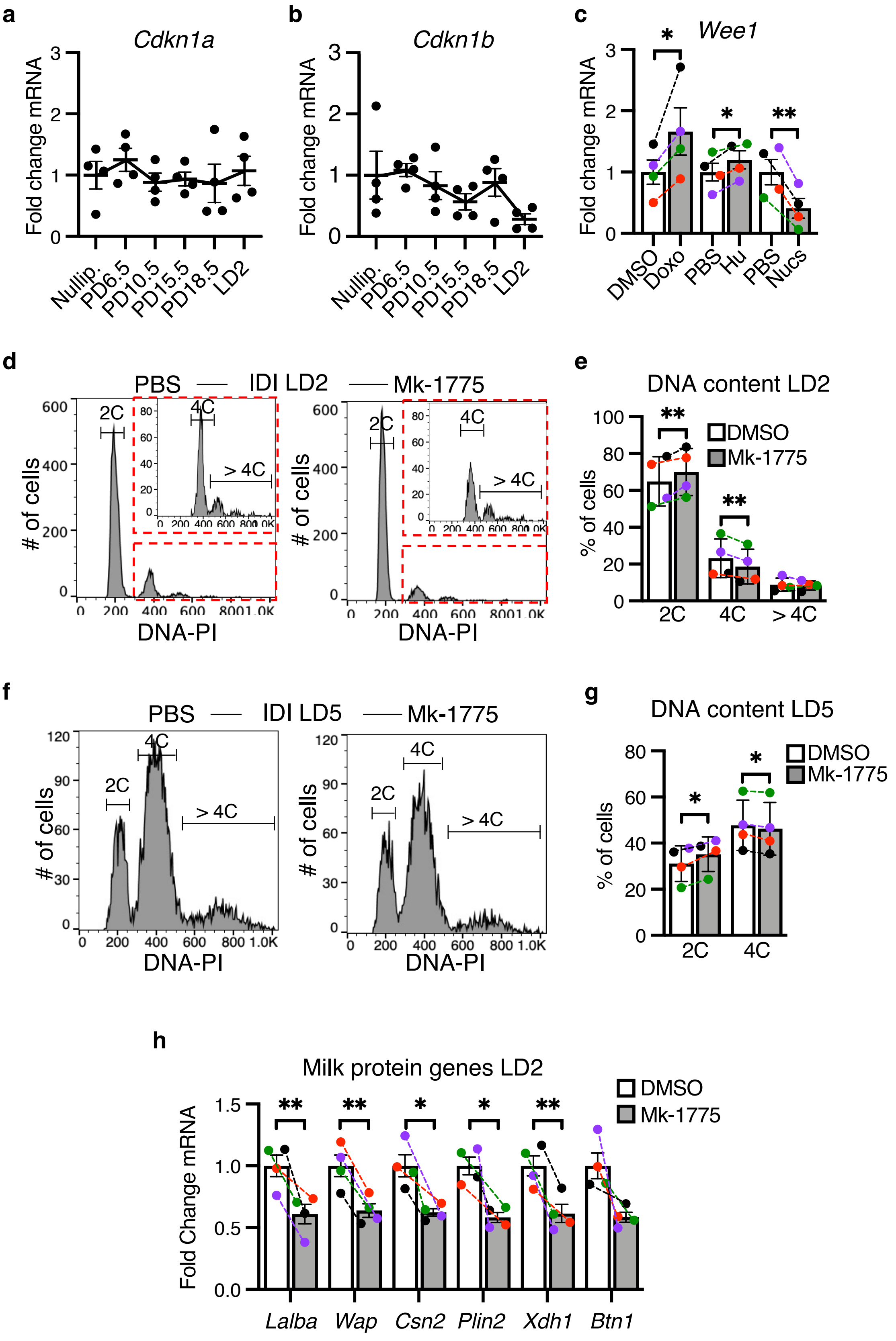
a
Cdkn1a
Fold change mRNA
Nullip.
PD6.5
PD10.5
PD15.5
PD18.5
LD2
b
Cdkn1b
Fold change mRNA
c
Wee1
Fold change mRNA
DMSO
Doxo
PBS
Hu
PBS
Nucs
d
PBS — IDI LD2 — Mk-1775
# of cells
2C
4C
> 4C
DNA-PI
e
DNA content LD2
DMSO
Mk-1775
% of cells
2C
4C
> 4C
f
PBS — IDI LD5 — Mk-1775
# of cells
2C
4C
> 4C
DNA-PI
g
DNA content LD5
DMSO
Mk-1775
% of cells
2C
4C
h
Milk protein genes LD2
DMSO
Mk-1775
Fold Change mRNA
Lalba
Wap
Csn2
Plin2
Xdh1
Btn1

Supplementary Figure 9. The DNA damage response to replication stress regulates endoreplication through WEE1. a and b) *Cdkn1a* (**a**) and *Cdkn1b* (**b**) expression in whole tissue from nulliparous, PD6.5, PD10.5, PD15.5, PD18.5 and LD2 MGs, as detected by RT-qPCR. **c)** *Wee1* expression in whole MG tissue after contralateral IDI with DMSO/Doxo, PBS/Hu or PBS/Nucs, as detected by RT-qPCR. Colored data points and dashed lines represent paired samples. **d)** Representative FACS DNA content analysis histograms of CK8$^+$ cells from LD2 MGs after contralateral IDI with DMSO or Mk-1775. Red dashed lines show a magnification of the histogram corresponding to the 4C and >4C DNA content populations. **e)** Percentage of CK8$^+$ cells with 2C, 4C or > 4C DNA content in LD2 MGs, as detected by FACS analysis, after contralateral IDI with DMSO or Mk-1775. Colored data points and dashed lines represent paired samples. **f)** Representative FACS DNA content analysis histograms of CK8$^+$ cells from LD5 MGs after contralateral IDI with DMSO or nucleosides Mk-1775. Red dashed lines show a magnification of the histogram corresponding to the 4C and >4C DNA content populations. **g)** Percentage of CK8$^+$ cells with 2C or 4C DNA content in LD5 MGs, as detected by FACS analysis, after contralateral IDI with DMSO or Mk-1775. Colored data points and dashed lines represent paired samples. **h)** Milk protein gene expression, *Lalba, Wap, Csn2, Plin2, Xdh1* and *Btn1*, expression in LD2 MGs after contralateral IDI with DMSO or Mk-1775, as detected by RT-qPCR. Colored data points and dashed lines represent paired samples. Data presented as mean ± SEM **(a-c and h)** and mean ± SD **(e and g)**. Data analyzed by one-way ANOVA with Tukey's multiple comparison test **(a and b)** and paired, two-tailed Student's t-test **(c, e, g and h)**. Data representative of n=4 biologically independent experiments. p values: * < 0.05, **< 0.01.

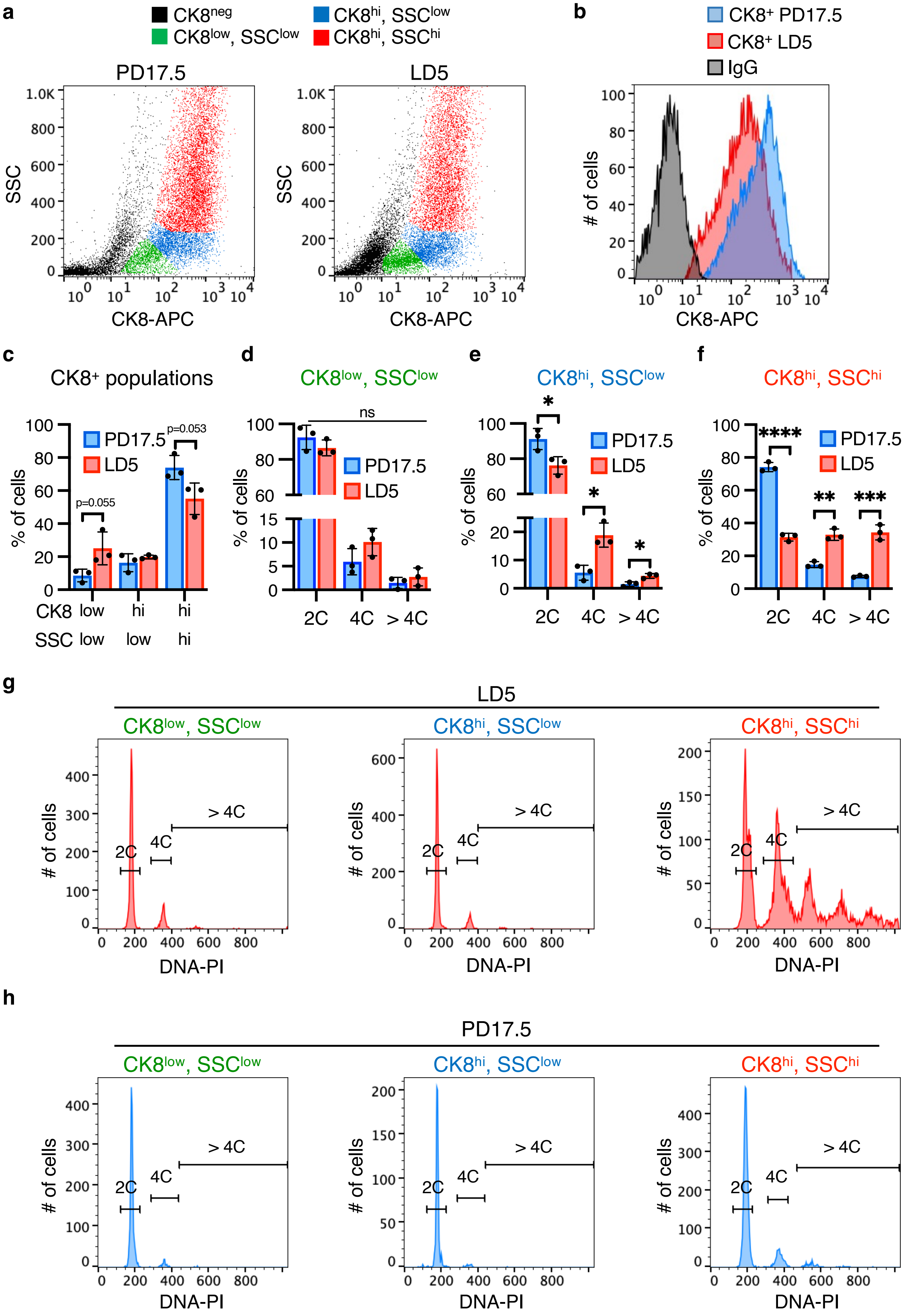
a
CK8neg
CK8low, SSClow
CK8hi, SSClow
CK8hi, SSChi
PD17.5
LD5
SSC
CK8-APC
b
CK8+ PD17.5
CK8+ LD5
IgG
# of cells
CK8-APC
c
CK8+ populations
PD17.5
LD5
p=0.053
p=0.055
% of cells
CK8 low hi hi
SSC low low hi
d
CK8low, SSClow
ns
PD17.5
LD5
% of cells
2C 4C > 4C
e
CK8hi, SSClow
PD17.5
LD5
% of cells
2C 4C > 4C
f
CK8hi, SSChi
PD17.5
LD5
% of cells
2C 4C > 4C
g
LD5
CK8low, SSClow
CK8hi, SSClow
CK8hi, SSChi
# of cells
2C
4C
> 4C
DNA-PI
h
PD17.5
CK8low, SSClow
CK8hi, SSClow
CK8hi, SSChi
# of cells
2C
4C
> 4C
DNA-PI

Supplementary Figure 10. Tetraploid and polyploid cells emerge in a CK8^{high} luminal subpopulation at LD5. a) Representative dot plots showing the CK8 negative (black), CK8^{low}; SSC^{low} (green), CK8^{high}; SSC^{low} (blue) and CK8^{high}; SSC^{high} (red) populations, as detected by FACS analysis, from PD17.5 (left) or LD5 (right) MGs. **b)** CK8 expression in PD17.5 (blue) or LD5 (red) whole mammary gland (MG) cell preps. CK8^{+} cells were detected by using an IgG negative control (grey). **c)** Percentage of CK8^{low}; SSC^{low}, CK8^{high}; SSC^{low} and CK8^{high}; SSC^{high} cells from PD17.5 (blue) or LD5 (red) MGs, as detected by FACS analysis. **d-f)** Percentage of cells with 2C, 4C and >4C DNA content within the CK8^{low}; SSC^{low} **(d)**, CK8^{high}; SSC^{low} **(e)** and CK8^{high}; SSC^{high} **(f)** populations, as detected by FACS analysis, from PD17.5 (blue) or LD5 (red) MGs. **g and h)** Representative FACS histograms for DNA content analysis of the CK8^{low}; SSC^{low}, CK8^{high}; SSC^{low} and CK8^{high}; SSC^{high} populations from LD5 **(g)** or PD17.5 **(h)** MGs. Data presented as mean ± SD. Data analyzed by unpaired, two-tailed Student's t-test. Data representative of n=3 biologically independent experiments. p values: * < 0.05, **< 0.01, *** < 0.001, **** < 0.0001.

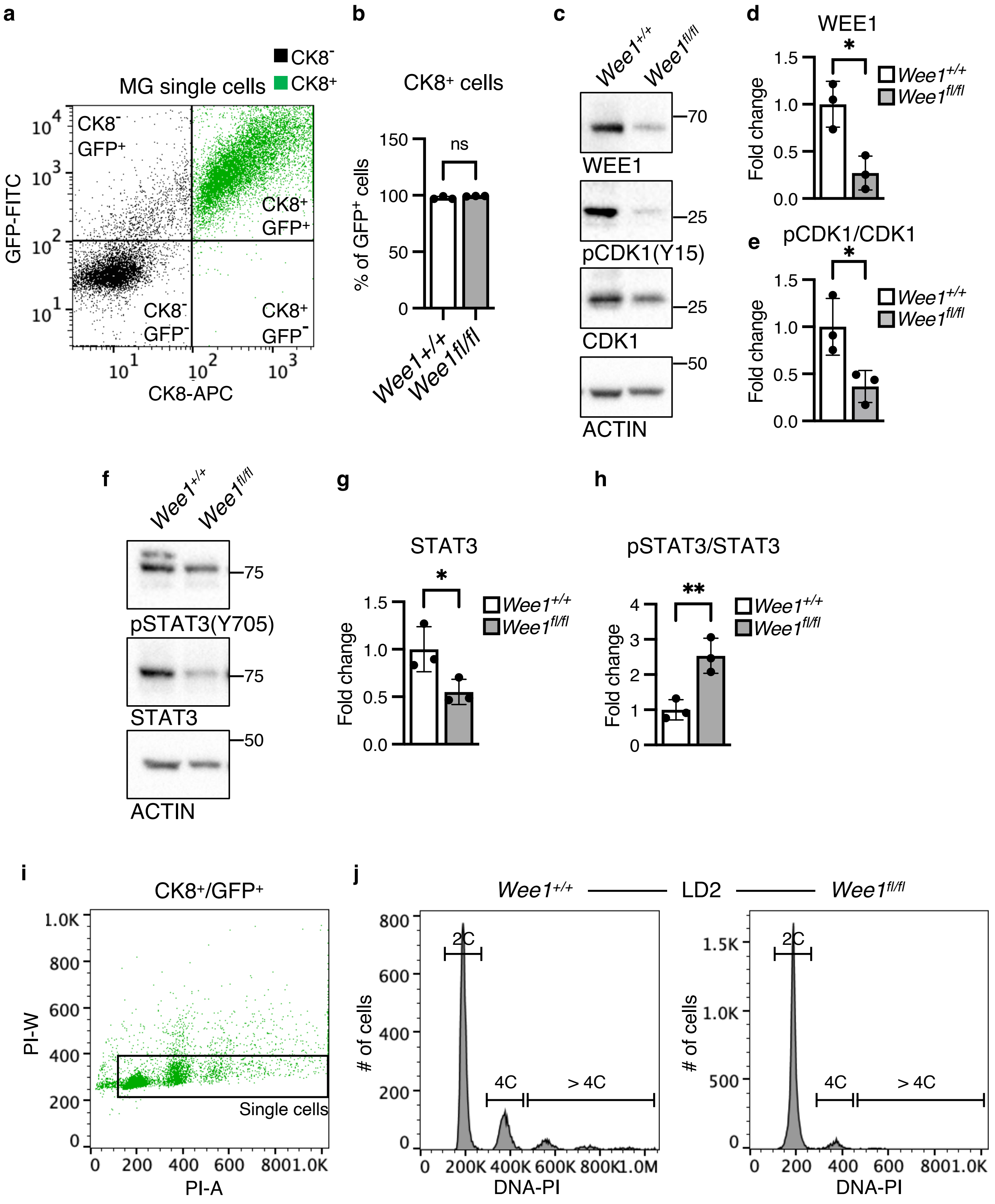

a
MG single cells
CK8-
CK8+
CK8- GFP+
CK8+ GFP+
CK8- GFP-
CK8+ GFP-
GFP-FITC
CK8-APC
b
CK8+ cells
ns
% of GFP+ cells
Wee1+/+
Wee1fl/fl
c
Wee1+/+
Wee1fl/fl
WEE1
pCDK1(Y15)
CDK1
ACTIN
70
25
25
50
d
WEE1
Fold change
e
pCDK1/CDK1
Fold change
f
pSTAT3(Y705)
STAT3
ACTIN
75
75
50
g
STAT3
Fold change
h
pSTAT3/STAT3
Fold change
i
CK8+/GFP+
PI-W
PI-A
Single cells
j
Wee1+/+
LD2
Wee1fl/fl
# of cells
DNA-PI
2C
4C
> 4C

Supplementary Figure 11. WEE1 regulates alveolar endoreplication during lactation. **a)** Representative dot plot of CK8 and GFP expression in *Wee* $^{fl/fl}$ mammary cells at LD2, as detected by FACS analysis. **b)** Percentage of $CK8^+$ cells from *Wee1*$^{+/+}$ and *Wee* $^{fl/fl}$ MGs at LD2 that are GFP^+, as detected by FACS analysis. **c-e)** Representative western blot **(c)** and quantifications of WEE1 **(d)**, pCDK1(Y15) and CDK1 **(e)** expression in *Wee1*$^{+/+}$ and *Wee* $^{fl/fl}$ MGs at LD2. Quantification **(e)** shown as pCDK1/CDK1 ratio. **f-h)** Representative western blot **(f)** and quantifications of STAT3 **(g)** and pSTAT3 (Y705) **(h)** expression in *Wee1*$^{+/+}$ and *Wee* $^{fl/fl}$ MGs at LD2. Quantification **(h)** shown as pSTAT3/STAT3 ratio. **i)** Representative dot plot of MG $CK8^+$/GFP^+ cells showing the identification of single cells, based on propidium iodide area (PI-A) versus width (PI-W). Black box shows the gating strategy for single cell identification. **j)** Representative FACS DNA content analysis histograms from $CK8^+$ *Wee1*$^{+/+}$ and *Wee* $^{fl/fl}$ MGs at LD2. Data presented as mean ± SD. Data analyzed by unpaired, two-tailed Student's t-test. Data representative of n=3 biologically independent experiments. p values: p values: * < 0.05, **< 0.01.
