# Supplementary material for: Physiological DNA damage promotes functional endoreplication of mammary gland alveolar cells during lactation
Source: Nat Commun. 2024 Apr 17;15:3288. doi: 10.1038/s41467-024-47668-9 (PMC11021458; doi:10.1038/s41467-024-47668-9)
Supplement: Supplementary file 3 — Reporting Summary [file 41467_2024_47668_MOESM3_ESM.pdf]

nature portfolio

Corresponding author(s): Lindsay Hinck, NCOMMS-22-27547B

Last updated by author(s): Mar 28, 2024

Reporting Summary

Nature Portfolio wishes to improve the reproducibility of the work that we publish. This form provides structure for consistency and transparency in reporting. For further information on Nature Portfolio policies, see our Editorial Policies and the Editorial Policy Checklist.

Statistics

For all statistical analyses, confirm that the following items are present in the figure legend, table legend, main text, or Methods section.

n/a	Confirmed	
[ ]	[x]	The exact sample size (n) for each experimental group/condition, given as a discrete number and unit of measurement
[ ]	[x]	A statement on whether measurements were taken from distinct samples or whether the same sample was measured repeatedly
[ ]	[x]	The statistical test(s) used AND whether they are one- or two-sided<br>*Only common tests should be described solely by name; describe more complex techniques in the Methods section.*
[x]	[ ]	A description of all covariates tested
[x]	[ ]	A description of any assumptions or corrections, such as tests of normality and adjustment for multiple comparisons
[x]	[ ]	A full description of the statistical parameters including central tendency (e.g. means) or other basic estimates (e.g. regression coefficient) AND variation (e.g. standard deviation) or associated estimates of uncertainty (e.g. confidence intervals)
[x]	[ ]	For null hypothesis testing, the test statistic (e.g. F, t, r) with confidence intervals, effect sizes, degrees of freedom and P value noted<br>*Give P values as exact values whenever suitable.*
[x]	[ ]	For Bayesian analysis, information on the choice of priors and Markov chain Monte Carlo settings
[x]	[ ]	For hierarchical and complex designs, identification of the appropriate level for tests and full reporting of outcomes
[x]	[ ]	Estimates of effect sizes (e.g. Cohen's d, Pearson's r), indicating how they were calculated

Our web collection on statistics for biologists contains articles on many of the points above.

Software and code

Policy information about availability of computer code

Data collection	Microscopy data were collected using Zeiss Zen (black v.2.3 SP1) and Zeiss Zen Pro (blue v.3.6) software. RT-qPCR data were collected using Bio-Rad CFX Manager (v.3.1) software. Western blot data were collected using Bio-Rad Image Lab (v.6.0.1) software. Flow cytometry data were collected using Beckman Coulter CytExpert (v.2.4) and BD FACSDiva (v.8.0.1) software.
Data analysis	Microscopy analyses were performed using ImageJ2 (v.2.14) and CellProfiler (v.4.2.1) software. Flow cytometry analyses were performed using FlowJo (v.10.9.0) software. RT-qPCR analyses were performed using Microsoft Office Excel (v.16) software. Data representation and statistical analyses were performed using GraphPad Prism (v.9) software.

For manuscripts utilizing custom algorithms or software that are central to the research but not yet described in published literature, software must be made available to editors and reviewers. We strongly encourage code deposition in a community repository (e.g. GitHub). See the Nature Portfolio guidelines for submitting code & software for further information.

Data

Policy information about availability of data

All manuscripts must include a data availability statement. This statement should provide the following information, where applicable:

- Accession codes, unique identifiers, or web links for publicly available datasets
- A description of any restrictions on data availability
- For clinical datasets or third party data, please ensure that the statement adheres to our policy

The primary data generated in this study are provided in the Source Data file. Any additional data are available from the corresponding author upon request.

Research involving human participants, their data, or biological material

Policy information about studies with human participants or human data. See also policy information about sex, gender (identity/presentation), and sexual orientation and race, ethnicity and racism.

Reporting on sex and gender	N/A
Reporting on race, ethnicity, or other socially relevant groupings	N/A
Population characteristics	N/A
Recruitment	N/A
Ethics oversight	N/A

Note that full information on the approval of the study protocol must also be provided in the manuscript.

Field-specific reporting

Please select the one below that is the best fit for your research. If you are not sure, read the appropriate sections before making your selection.

☒ Life sciences ☐ Behavioural & social sciences ☐ Ecological, evolutionary & environmental sciences

For a reference copy of the document with all sections, see nature.com/documents/nr-reporting-summary-flat.pdf

Life sciences study design

All studies must disclose on these points even when the disclosure is negative.

Sample size	No sample size calculation was performed. For experiments using animals, in accordance with the guidelines set by the University of California, Santa Cruz (UCSC) Institutional Animal Care and Use Committee (IACUC), reduction of animal use was a priority such that 2 < n < 5 biological replicates was chosen.
Data exclusions	Data were only excluded if identified as an outlier using the Robust Regression & Outlier Removal (ROUT) method with a coefficient of Q=1% in GraphPad Prism.
Replication	All reasonable measures were taken to ensure the reproducibility of experimental findings. Such measures include the use of independently collected tissues and cells for biological replicates. All attempts to verify reproducibility were successful. A minimum of three independent experiments were performed and represented in all figures.
Randomization	Randomization is not relevant to the study. Samples were allocated into experimental groups based on treatment or genotype.
Blinding	Blinding was not relevant to the study as data collection and analyses are quantitative and not qualitative.

Reporting for specific materials, systems and methods

We require information from authors about some types of materials, experimental systems and methods used in many studies. Here, indicate whether each material, system or method listed is relevant to your study. If you are not sure if a list item applies to your research, read the appropriate section before selecting a response.

Materials & experimental systems

n/a	Involved in the study	
	x	Antibodies
	x	Eukaryotic cell lines
x		Palaeontology and archaeology
	x	Animals and other organisms
x		Clinical data
x		Dual use research of concern
x		Plants

Methods

n/a	Involved in the study	
x		ChIP-seq
	x	Flow cytometry
x		MRI-based neuroimaging

Antibodies

Antibodies used

Primary Antibodies:
Rat Anti-Cytokeratin 8 [Developmental Studies Hybridoma Bank, TROMA-I]
Rat Anti-E Cadherin [Invitrogen, 13-1900, Clone# ECCD-2]
Rabbit Anti-Histone H2A.X (Phospho-Ser139) [Cell Signaling Technology, 2577]
Rabbit Anti-53BP1 [Cell Signaling Technology, 4937]
Rabbit Anti-RPA32/RPA2 [Abcam, ab76420]
Rabbit Anti-ATR [Cell Signaling Technology, 2790]
Rabbit Anti-ATR (Phospho-Thr1989) [GeneTex, GTX128145]
Mouse Anti-ATM [GeneTex, GTX70103]
Rabbit Anti-ATM (Phospho-Ser1981) [GeneTex, GTX132146]
Mouse Anti-CHK1 [Santa Cruz Biotechnology, sc-8408]
Rabbit Anti-CHK1 (Phospho-Ser345) [Cell Signaling Technology, 2348]
Rabbit Anti-WEE1 [Invitrogen, PA5-29303]
Rabbit Anti-Mouse Milk Proteins [Accurate Chemical & Scientific, YNRMTM]
Rabbit Anti-CSN2 [Abclonal, A12749]
Rabbit Anti-PLIN2 [provided by Dr. James McManaman]
Mouse Anti-Cyclin B1 [Santa Cruz Biotechnology, sc-245]
Rabbit Anti-Cyclin E1 [Sigma Aldrich, SAB4503516]
Mouse Anti-CDC2 [Santa Cruz Biotechnology, sc-54]
Rabbit Anti-CDC2 (Phospho-Tyr15) [Cell Signaling Technology, 4539]
Rabbit Anti-STAT5 [Santa Cruz Biotechnology, sc-836]
Rabbit Anti-STAT5 (Phospho-Tyr694) [Cell Signaling Technology, 9351]
Mouse Anti-STAT3 [Cell Signaling Technology, 9139]
Rabbit Anti-STAT3 (Phospho-Tyr705) [Cell Signaling Technology, 9145]
Rabbit Anti-GFP [GenScript, A01704]
Mouse Anti-Beta Actin [Santa Cruz Biotechnology, sc-47778]
Mouse Anti-GAPDH [Santa Cruz Biotechnology, sc-365062]
Mouse Anti-HSP70 [Santa Cruz Biotechnology, sc-24]
Rabbit IgG Isotype Control [Invitrogen, 10500]
Rat IgG Isotype Control [Invitrogen, 10700]
Rat Anti-BrdU [Abcam, ab6326]

Secondary Antibodies:
Donkey Anti-Rabbit IgG HRP [Jackson ImmunoResearch, 711-035-152]
Donkey Anti-Mouse IgG HRP [Jackson ImmunoResearch, 715-035-150]
Donkey Anti-Rat IgG FITC [Invitrogen, A24544]
Donkey Anti-Rabbit IgG FITC [Invitrogen, A16030]
Donkey Anti-Rat IgG APC [Jackson ImmunoResearch, 712-136-153]
Donkey Anti-Rabbit IgG AlexaFluor 488 [Invitrogen, A32790]
Donkey Anti-Rabbit IgG AlexaFluor 647 [Invitrogen, A32795
Donkey Anti-Rat IgG AlexaFluor 488 [Invitrogen, A48269]
Donkey Anti-Rat IgG AlexaFluor 647 [Invitrogen, A48272]
Donkey Anti-Mouse IgG AlexaFluor 488 [Invitrogen, A32766]
Donkey Anti-Mouse IgG AlexaFluor 594 [Invitrogen, A32744]
Donkey Anti-Mouse IgG AlexaFluor 647 [Invitrogen, A32787]

Validation

Validation of each antibody is available on the website of its respective manufacturer. Antibodies were tested and titrated following the manufacturer's recommended dilution or concentration for each application, as noted on the manufacturer's website. Immunofluorescence and western blotting were performed using isotype and secondary-only negative controls, as well as plasmid over-expression positive controls when reasonably available. Flow cytometry was performed using isotype and fluorescence-minus-one (FMO) negative controls.

Eukaryotic cell lines

Policy information about cell lines and Sex and Gender in Research

Cell line source(s)	The HC11 cell line was obtained from American Type Culture Collection (ATCC, CRL-3062). All murine primary cells used for organoid culture were obtained from wildtype female CD-1 strain mice of the species Mus Musculus.
Authentication	The HC11 cell line was authenticated by ATCC using cell growth properties, cell morphology and Cytochrome C Oxidase I (COI) assay testing.
Mycoplasma contamination	All cell lines tested negative for mycoplasma contamination.
Commonly misidentified lines (See ICLAC register)	No commonly misidentified lines were used in this study.

Animals and other research organisms

Policy information about studies involving animals; ARRIVE guidelines recommended for reporting animal research, and Sex and Gender in Research

Laboratory animals	The study involved the use of the mouse species Mus Musculus. CD-1 strain mice were provided by Charles River Laboratories. C57BL/6J-Wee1<tm1.1 mrl> strain mice were generated and provided by Taconic. Ck8-CreER/mTmG strain mice, a cross of Tg(Krt8-cre/ERT2)17Blpn/J (JAX:017947) and B6.129(Cg)-Gt(ROSA)26Sortm4(ACTB-tdTomato, -EGFP)Luo/J (JAX: 007676) strains, were provided by Dr. Diwakar R Pattabiraman. All mice used in the study were 10-12 weeks old, except for lactation experiments in which newborn (1-5 days old) pups were also involved.
Wild animals	The study did not involve wild animals.
Reporting on sex	The findings of the study apply only to the female sex as the study pertains to mammary gland development during pregnancy and lactation.
Field-collected samples	The study did not involve field-collected samples.
Ethics oversight	All animal procedures were both approved by and conducted in accordance with the guidelines set by the University of California Santa Cruz (UCSC) Institutional Animal Care and Use Committee (IACUC).

Note that full information on the approval of the study protocol must also be provided in the manuscript.

Plants

Seed stocks	N/A
Novel plant genotypes	N/A
Authentication	N/A

Flow Cytometry

Plots

Confirm that:

- [x] The axis labels state the marker and fluorochrome used (e.g. CD4-FITC).
- [x] The axis scales are clearly visible. Include numbers along axes only for bottom left plot of group (a 'group' is an analysis of identical markers).
- [x] All plots are contour plots with outliers or pseudocolor plots.
- [x] A numerical value for number of cells or percentage (with statistics) is provided.

Methodology

Sample preparation	Mechanically dissociated inguinal, abdominal, and thoracic mammary fat pads were prepared into cell suspension for flow cytometry or fluorescence-activated cell sorting (FACS). The lymph node was removed from abdominal glands. Glands were chopped using a mechanical tissue chopper and digested for 1 h at 37°C in digestion media [RPM1 containing 1%FBS,

collagenase IA (Sigma, C9891), hyaluronidase (Sigma, H3506) and DNAse I (Worthington, LS002007)]. Tissue was washed with washing buffer (1X PBS containing 2% FBS) and centrifuged at 1,000 rpm for 5 min at 4°C. Tissue was further digested using pre-warmed 0.25% Trypsin-EDTA (Thermo Fisher, 25200056), washed, and digested with 5mg/ml of pre-warmed dispase II (Roche, 4942078001). Red blood cells were lysed using Ammonium Chloride Solution (Stem Cell Technologies, 07850). Cells were washed, resuspended and filter through a 70 µm cell strainer (Falcon, 08-771-2) and processed for downstream applications. For DNA content analysis, cells were fixed in ice-cold 70% EtOH at a final concentration of 1 million cells per millileter and vortexed vigorously to avoid cell aggregates. Cells were then washed twice with washing buffer [1X PBS containing 5% FBS and 0.5% tween 20 (Fisher Chemical, BP337500)] and centrifuged at 2,000 rpm for 5 min at 4°C. The pellet was resuspended for staining and analysis.

For DNA content analysis of HC11 cells, cells were washed with 1X DPBS (GIBCO, 14190-250) and harvested using 0.05% Trypsin-EDTA (GIBCO, 25300-062). Cell suspension was washed with 1X DPBS and centrifuged at 1,000 rpm for 5 min at 4°C. Cell pellet was fixed in ice-cold 70% EtOH and then vortexed vigorously to avoid cell aggregates. After fixation, cells were washed with washing buffer [1X PBS (GIBCO, 14190136) supplemented with 5% FBS] and centrifuged at 2,000 rpm for 5 min at 4°C. The pellet was resuspended for staining and analysis.

Instrument	Beckman Coulter Cytoflex LX, BD FACSAria IIU, BD LSR II
Software	Beckman Coulter CytExpert, BD FACSDiva
Cell population abundance	Populations sorted based on DNA content were re-analyzed by flow-cytometry to confirm their tetraploid (4C) or polyploid (>4C) status.
Gating strategy	For the analysis of primary cells, the luminal mammary gland population was identified based on the expression of Cytokeratin-8 (CK8). A negative isotype control was utilized for the identification of this population. For the analysis of both primary cells and HC11 cells, singlets were identified based on the PI-Area vs PI-Weight.

☒ Tick this box to confirm that a figure exemplifying the gating strategy is provided in the Supplementary Information.
